# Emerging Non-Canonical Functions and Regulation by p53: p53 and Stemness

**DOI:** 10.3390/ijms17121982

**Published:** 2016-11-26

**Authors:** David J. Olivos, Lindsey D. Mayo

**Affiliations:** 1 Department of Microbiology and Immunology, Indiana University School of Medicine, Indianapolis, IN 46202, USA; djolivos@iupui.edu; 2 Department of Pediatrics, Herman B Wells Center for Pediatrics Research, Indiana University School of Medicine, Indianapolis, IN 46202, USA; 3 Department of Biochemistry and Molecular Biology, Indiana University School of Medicine, Indianapolis, IN 46202, USA

**Keywords:** p53, mutant p53, normal stem cells, cancer stem cell (CSC), translational modifications, survival, colonization, Mdm2, non-canonical function, niche, regulation

## Abstract

Since its discovery nearly 40 years ago, p53 has ascended to the forefront of investigated genes and proteins across diverse research disciplines and is recognized most exclusively for its role in cancer as a tumor suppressor. Levine and Oren (2009) reviewed the evolution of p53 detailing the significant discoveries of each decade since its first report in 1979. In this review, we will highlight the emerging non-canonical functions and regulation of p53 in stem cells. We will focus on general themes shared among p53’s functions in non-malignant stem cells and cancer stem-like cells (CSCs) and the influence of p53 on the microenvironment and CSC niche. We will also examine p53 gain of function (GOF) roles in stemness. Mutant p53 (*mut*p53) GOFs that lead to survival, drug resistance and colonization are reviewed in the context of the acquisition of advantageous transformation processes, such as differentiation and dedifferentiation, epithelial-to-mesenchymal transition (EMT) and stem cell senescence and quiescence. Finally, we will conclude with therapeutic strategies that restore wild-type p53 (*wt*p53) function in cancer and CSCs, including RING finger E3 ligases and CSC maintenance. The mechanisms by which *wt*p53 and *mut*p53 influence stemness in non-malignant stem cells and CSCs or tumor-initiating cells (TICs) are poorly understood thus far. Further elucidation of p53’s effects on stemness could lead to novel therapeutic strategies in cancer research.

## 1. Introduction

### 1.1. The p53 Network and Its Role in Cancer

Our knowledge of p53 has developed extensively over the past four decades as scientists have unraveled its distinct biological processes. p53 has been associated with several functions in normal cells and cancer. The discoveries by six independent laboratories in 1979 have become the focal point igniting what is now decades of research devoted to p53. These studies found the 53-kDa protein to be bound to Large Tumor Antigen in Simian Virus (SV40) infected tumor cells. In the same period, a separate group identified p53 in transformed but not normal mouse cells from reactions with mouse sarcoma antiserum [1,2,3,4,5,6]. Initially, p53 was thought to be an oncogene [6,7,8]; however, the consensus in the next decade acknowledged the main function of p53 as a tumor suppressor that is often mutated or nonfunctional in cancer [9,10,11,12,13]. In contrast to *mut*p53, overexpression of *wt*p53 halted cell proliferation through control of the cell cycle and induction of apoptosis. In fact, in leukemia cells expression of *wt*p53 led to apoptosis confirming the tumor suppressor function of *wt*p53 [14,15,16,17]. Dysfunctional regulation at cell cycle checkpoints that are normally regulated by *wt*p53, are bypassed by damaged cells when p53 is inactivated or mutated leading to uncontrolled growth and carcinogenesis [18,19].

**Figure 1:**
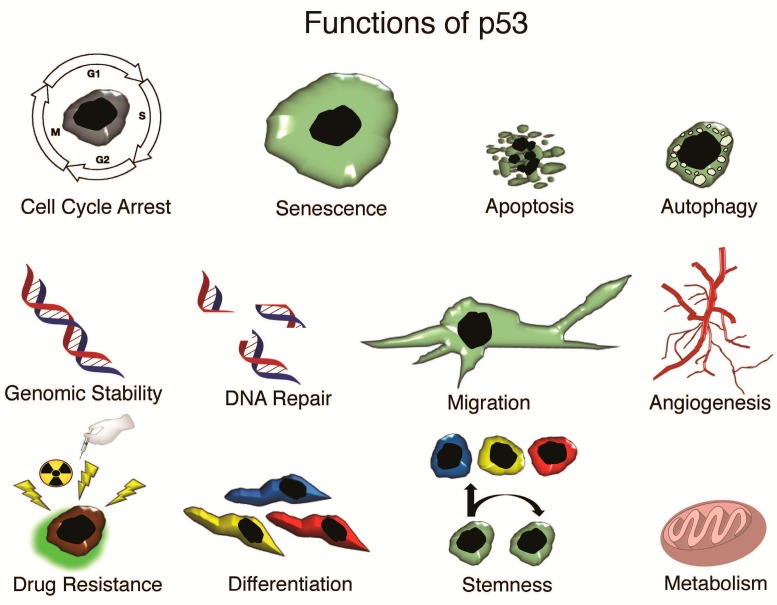
Functions of p53.

Most of the investigated functions of p53 are associated with full-length p53 (TAp53). Twelve isoforms are encoded in the TP53 gene: TAp53 (α,β,γ) ∆40p53 (α,β,γ), ∆133p53 (α,β,γ) and ∆160p53 (α,β,γ) [20,21,22,23]. The transactivation domain is present in long p53 isoforms TAp53 and ∆40p53, but not short p53 isoforms ∆133p53 and ∆160p53. Isoforms β and γ do not contain a canonical C-terminal oligomerization domain, but rather possess an additional domain with unknown function(s) [24]. The most prominent activity associated with p53 is its function as a tumor suppressor. As the “guardian of the genome”, p53 induces cell cycle arrest, apoptosis, autophagy or senescence in response to DNA damage and other genotoxic insults. p53 restricts cell proliferation to limit the continual propagation of abnormal genomes pillaged by ribonucleotide depletion, nutritional starvation, and hypoxia. Additionally, p53 has been shown to regulate microRNAs, energy metabolism, and anti-oxidant defense [25,26,27,28] (Figure 1).

In response to these stress factors, p53 is stabilized through posttranslational modifications via phosphorylation, acetylation, ubiquitination, methylation, neddylation, or SUMOylation [26,27,28,29]. For example, p53 is stabilized by the phosphorylation of the N-terminus at amino acid sites Ser^15^, Ser^20^, Ser^33^, Ser^37^, Ser^46^, Thr^18^ and Th^81^, which block the Mdm2 binding to p53. The C-terminus of p53 can be phosphorylated to activate p53 DNA binding activity at Ser^315^ and Ser^392^. Acetylation at Lys^320^, Lys^373^ and Lys^382^ or SUMOylation at Lys^386^ has also been observed in this region [30,31]. Once activated the interactive p53 network seeks to maintain genetic stability and prevent tumorigenesis. Likewise, p53 levels and activity must be tightly controlled to permit the growth and development of normal cells. p53 can upregulate the Mdm2 gene, while elevated levels of p53 can signal Mdm2 downregulation of p53 via ubiquitination and proteosomal degradation maintaining normal p53 steady state levels through a negative feedback loop [32,33,34,35]. Genotoxic stress, such as radiation exposure can increase p53 levels and transcriptional activity resulting in the inhibition of DNA replication to allow for DNA repair or enable cell death processes to prevent the continual propagation of malignant cells. DNA damage such as double-strand breaks can activate upstream mediators including protein kinases ATM, ATR, Chk1, and Chk2 leading to phosphorylation of p53 and Mdm2. These post-translational modifications interfere with the formation of the p53-Mdm2 complex resulting in p53 stabilization, increased p53 protein levels and transcriptional activity, and activation of cell cycle arrest, senescence, DNA repair and apoptosis [19,25,36].

In another example of p53 regulation through a negative feedback loop, ubiquitin ligases Cop1 and Pirh2 associate with p53 before undergoing ubiquitination and proteosomal degradation [37,38]. The tumor suppressor PTEN can lead to activation of p53 through the downregulation of Mdm2 [39,40,41]. Additionally, Dapk1, c-Ha-Ras and DDR1 form positive feedback loops on p53 that can activate p19ARF to inhibit Mdm2 [42,43,44]. Likewise, p21 inhibits cyclinE-cdk2 and increases formation of the Rb-Mdm2 complex and activation of p53 [39]. Cyclin G, another p53 target protein, can recruit protein phosphatase 2A (PP2A) and lead to Mdm2 and ATM dephosphorylation [45,46]. Furthermore, p53 activity is regulated by interactions with Wnt/β-catenin, IGF-1-AKT, Rb-E2F, p14/19ARF, cyclin-cdk, p38 MAP kinase, cyclin G-PP2A and p73 [39].

p53 mutations generated by missense mutations in the TP53 gene have been implicated in tumor progression as a result of *wt*p53 loss of functions as a tumor suppressor. Similarly, tumor-associated *mut*p53 acquires gain-of-function (GOF) oncogenic properties that are independent of *wt*p53. These mutations enable cancer-associated *mut*p53 with new properties that benefit the tumor at various levels of cancer progression. *Mut*p53 can exert its GOF properties through binding and inactivation of p53 associated proteins such as p53 family members p63 and p73. *Mut*p53 GOF processes are associated with a variety of physiological events: tumor cell proliferation, invasion, survival, metabolic changes, angiogenesis, metastasis, tissue modeling, enhanced chemoresistance, mitogenic defects, genomic instability and stem cell expansion [47,48,49,50,51]. Most p53 mutations associated with cancer are present in the p53 DNA binding domain (DBD) and are characterized as DNA contact mutants (hotspot mutant p53R273H) or conformational mutants (hotspot mutant p53R175H) [52]. *Mutp53* has been shown to upregulate numerous genes, including MDR1, PCNA, EGFR, IGF1R, c-myc and IGF2 [53,54,55,56,57,58]. *Mut*p53 can also downregulate pro-apoptotic genes such as caspase-3, and CD95/Fas/Apo1, and *wt*p53 target genes including p21 and PTEN [59,60,61,62].

The p53 family includes p63 and p73, which share similar gene structures and overlapping, but distinct functions cover a wide range of processes involved in development, apoptosis and cell cycle arrest, chemosensitivity and immortalization [31]. p63 isoforms include TAp63 (α,β,γ) and Np63 (α,β,γ), while p73 C- and N-terminal isoforms include TA73 (α, β, γ, δ, ε, ζ, η and splice variants ∆2, ∆3, ∆2/3). Additionally, internal promoter variants include ∆Np73 (α, β, γ, δ, ε, ζ, η) [63,64,65,66,67]. In response to stress, p53 activates cell cycle arrest and regulates genes, such as p21 to halt cell proliferation; however, in the induction of apoptosis, p53 requires p63 and p73 to activate apoptotic gene promoters. In normal development, p63 is involved in forming teeth, hair and glands of the mammary and prostate. p73 is also involved in normal development, including neurogenesis, and pheromonal signaling [30]. The tumor suppressors p53, p63 and p73 regulate miRNAs that are critical to tumor inhibition and downregulation of EMT, metastasis, and the proliferation of CSCs [68].

### 1.2. Stemness in Normal and Cancer Cells

Stem cells are a rare population of cells with the innate capacity to self-renew and give rise to a lineage of progenitor cells that can mature and propagate into a variety of cell types. Residing in specific tissues, stem cells provide functions critical to the needs of their location while mediating homeostasis, maintenance and repair [69,70,71,72]. Three major stem cell classes include embryonic stem (ES) cells, adult (somatic, postnatal) stem cells and cancer stem-like cells (CSCs), and their states are generally described in increasing levels of differentiation: totipotent, pluripotent, multipotent and unipotent. Totipotent stem cells possess an unlimited capacity with the potential to develop into virtually any cell found in the body, while pluripotent stem cells have limited potential to differentiate into three embryonic lineages (ectoderm, mesoderm and endoderm). Further differentiated are lineage-restricted multipotent stem cells (lineage-directed progenitor cells) with limited ability to differentiate or to give rise to other cell types, whereas unipotent stem cells can only give rise to one type of cell. ESCs originate from mammalian embryos preimplantation, while adult stem cells or somatic cells represent the self-renewing cells in skin, hair, intestine, liver and hematopoietic tissues.

CSCs or tumor-initiating cells (TICs), are a rare population of cells with the ability to initiate the growth and maintenance of a heterogeneous tumor. They have been identified in a variety of hematopoietic cancers, solid tumors and tissues, including those of the bladder, breast, brain, lung, cervix and prostate [73,74,75,76,77]. Recent studies have demonstrated CSC frequencies to be not as scarce (0.0001% to 0.1%) as once believed, but that CSC numbers may be more dependent on cell type, stage of malignant progression, tumor cell phenotypic switching and even environmental factors, including foreign environments, such as those used in xenotransplantation assays [73,78,79,80,81,82,83,84]. In the case of xenotransplantation, Quintana et al. (2008) [85] demonstrated that NOD/SCID mice are prone to an underestimation of the frequency of tumorigenic human cancers. Using modified xenotransplantation assay conditions and highly immunocompromised (lacking mature T and B cells and functional NK cells) NOD/SCID interleukin-2 receptor gamma chain null (Il2rg^−/−^) mice, an increase in the detection of tumorigenic melanoma cells by several orders of magnitude was observed. Limiting dilution assays using unselected patient primary and metastatic melanoma cells from 12 patients revealed that 25% resulted in tumor formation, while tumors formed from 27% using unselected cells from four patients. Kelly et al. (2007) [80] suggested that the low frequency of tumor-sustaining cells could be associated with the inability of human tumor cells to adapt to the mouse microenvironment.

The origins of CSCs have not been completely revealed. However, it has been proposed that CSCs are long-lived adult stem cells or progenitors with accumulated neoplastic transformations that have enabled them to sustain cancer growth, progression and metastasis [86,87,88]. In lung cancer, for example, epithelial stem cells undergo malignant transformation [89]. A dysfunctional environment niche may also transform normal stem cells into CSCs. Houston et al. (2014) [90,91] demonstrated that bone marrow-derived cells recruited into gastric glands, upon chronic injury, develop into gastric adenocarcinoma. It has also been suggested that CSCs, or even recurrent CSCs (rCSCs), can arise through cell fusion between mutated stem cells and differentiated cells [87,92,93,94,95].

Besides tumor initiation and cancer relapse, CSCs have been purported to be a cause of metastasis. Due to the variety of CSC properties, subpopulations have been proposed to include primary CSCs (pCSCs) that induce tumor formation and metastatic CSC (mCSCs) that induce metastases [79]. The frequencies of CSCs in tumors are higher than non-malignant stem cells in normal tissues. The ability of stem cells to undergo asymmetrical and symmetrical division enable deregulated signaling pathways associated with self-renewal and stem cell development to control tumor progression, metastasis and recurrence. Hence, these normal stem cell development pathways (i.e., Notch and Wnt/β-catenin) may be manipulated to prevent CSC genesis [93,94,95,96,97,98,99,100].

Stem cells and cancer cells have a highly proliferative potential giving rise to normal and malignant heterogeneous tissues with cells exhibiting unique clonogenic properties [101,102,103]. According to the CSC theory, only a low percentage of unique cells are capable of initiating and maintaining a tumor [103]. Non-malignant tissues possess normal homeostatic mechanisms, unlike tumors that experience a deregulation of self-renewal [98,99]. CSCs identified from human brain and colon cancer express CSC cell surface marker CD133 and have the capacity to self-renew and differentiate, while CD133-tumor cells lack tumorigenicity [75,76,77]. Through transcriptional repression of CD133, *wt*p53 can inhibit CSCs. p53 recruits histone deacetylase 1 (HDAC1) to the CD133 promoter and reduces histone H3 acetylation. Reduction in CD133 expression inhibits tumor cell proliferation, colony formation and the expression of Nanog, Oct4, Sox2 and c-Myc stemness transcription factors [104].

Transcription factors serve critical functions in ESC self-renewal, and many are overexpressed in tumors. Somatic cell reprogramming into iPSCs can be initiated by epigenetic changes and the expression of transcription factors Sox-2, c-Myc, Oct-4, Klf-4 and Lin-28 [105]. Also associated with this programming is the ESC and inner cell mass (ICM) pluripotency factor Nanog [106]. After DNA damage, p53 binds to the Nanog promoter and suppresses Nanog expression through Ser215 p53 phosphorylation and p53 transcriptional activity [107]. Golubovskaya et al. (2013) [108] described the cross-talk signaling between focal adhesion kinase (FAK), p53, Mdm2 and Nanog in cancer stem cells. FAK binds and phosphorylates Mdm2, activates p53 degradation and has been implicated in CSC proliferation, motility, invasion and differentiation [109].

Since CSCs behave similarly to non-malignant stem cells, it is not surprising that *mut*p53 plays a role in this population as *wt*p53 is important to non-malignant stem cells. CSCs are less susceptible to chemotherapy compared to the vast majority of the tumors due to a slow proliferation rate, efficient DNA repair, high expression of anti-apoptotic proteins and ABC multi-drug pumps [110,111,112,113]. Interestingly, p53 splice variant isoform ∆133p53β promotes CSC potential in MCF-7 breast cancer cells, stimulating the expression of key pluripotency factors SOX2, OCT3/4 and Nanog [24,114].

### 1.3. Tumor Microenvironment and the CSC Niche

Stem cell processes are controlled by intrinsic factors that are cell-autonomous transcription factors and genes and a network of extrinsic factors that include soluble factors and adhesion molecules from ECM and niche cells [115]. Stem cells reside in niches or specialized microenvironments consisting of a symphony of interactive cells, including fibroblasts, endothelial and perivascular cells, adipose cells, cytokines, immune cells, macrophages, extracellular matrix (ECM) and soluble factors excreted from cells [116,117,118]. The interactions between stem cells and the niche play an important role in stem cell maintenance with CSCs driving niche formation and the niche controlling CSC self-renewal, proliferation, differentiation, invasion and metastasis [119,120,121,122,123,124,125]. The stem cell niche is regulated by extrinsic stimulation by Notch, Wnt, Hedgehog or BMP. The niche is specialized in preventing differentiation, maintaining CSC stemness and environments suitable for tumor survival and progression [126]. The niche is also an interactive network that is capable of metabolic sensing and mechanical inputs. Through adhesion molecules and paracrine factors, stem cells within the niche interact with supporting cells forming a complex network. Cell contact signals and exchanges in molecular signals help maintain the niche and support the unique characteristics of stem cells (pluripotency and self-renewal). Hypoxic sites in tumors can produce CSC niches and induce stemness in tumor cells through hypoxia inducible factor 1 (HIF-1) and activation of transcription factors involved in reprogramming of iPSCs: Oct4, Sox2, Nanog and KLF4 [127]. Hypoxic microenvironments also lead to increased VEGF and E-cadherin levels [128].

The tissue microenvironment is a powerful line of defense against tumorigenesis. Embryonic skin in the uterus can inhibit tumor formation of implanted murine B16 malignant melanoma cells. Likewise, chick and zebrafish embryonic microenvironments have been observed to inhibit tumor formation of implanted cancer cells [129,130,131,132]. The normal tissue microenvironment can be influenced and transformed into a malignant niche by cancer cells and the supporting stroma or accessary cells. In melanoma, endothelial cells and fibroblasts secrete factors that facilitate melanocyte cell movement from the basement membrane while accelerating the tumorigenic transformation of melanocytes [128,133,134]. Cancer-associated fibroblasts (CAFs) secrete hepatocyte growth factor and TGF-β, which mediate mutant p53-dependent invasion and metastasis. *mut*p53, through prolonging of NF-κB activation and cell survival, can promote the inflammatory environment needed for the development of colorectal tumors. Ubertini et al. (2015) [135] reported a novel *mut*p53 GOF in promoting inflammatory signals by the repression of the secreted interleukin-1 receptor antagonist (sIL-1Ra). The findings demonstrate that *mut*p53 tumorigenesis requires sIL-1Ra suppression, resulting in a chronic pro-inflammatory tumor microenvironment. *mut*p53 influences tumor and microenvironment interactions and may induce pro-oncogenic GOF changes in tumors and stromal cells alike. As demonstrated by Addadi et al. (2010) [136], *mut*p53-expressing fibroblasts could promote tumor growth better than p53 null fibroblasts.

*Mut*p53 GOF activities have been identified in cell reprogramming, expansion, maintenance and interaction with the tumor stroma. It is well known that *wt*p53 is a suppressor of somatic stem cell reprogramming, preventing differentiated somatic cells from being reprogrammed into pluripotent stem cells with unlimited expansion ability. *Mut*p53 can initiate tumor formation by promoting the generation and expansion of pluripotent cells. For example, loss of p53 results in the de-differentiation of somatic cells, while *mutp53* enhances reprogramming [20,137,138,139]. Hanel et al. (2013) [140] reported that *mut*p53 R238Q transgenic mice exhibited an expansion in HSC and mesenchymal stem cell (MSC) progenitors. Similar to cancer cells, CSCs may modulate the microenvironment through cell-to-cell contact or the secretion of factors. Topczewska et al. (2006) [141] showed that embryonic and tumorigenic pathways converge via Nodal signaling. Aggressive melanoma cells were injected into zebrafish blastula and were found to secrete embryonic morphogen Nodal, inducing the formation of ectopic embryonic axes. These findings demonstrate the potential of the microenvironment to drive and maintain tumorigenesis, including CSCs.

## 2. p53 and Stem Cells

Recent studies have implicated p53 in stem cell plasticity in the areas of self-renewal, differentiation and reprogramming and have associated these processes with tumor cell heterogeneity and progression. For example, focal adhesion kinase (FAK), p53 and Nanog have been found to be interlinked and to cross-regulate CSCs. FAK binds to Mdm2 and activates p53 degradation, whereas p53 upregulation inhibits Nanog and FAK survival signaling pathways, increasing CSC maintenance, cell growth and tumor inhibition [109]. Loss of p53 can induce Hedgehog signaling, upregulating Nanog in glial stem cells and CSCs. Po et al. (2010) [142] demonstrated that perturbing Hedgehog signaling alters the fate of Nanog to regulate self-renewal associated with glioma stem cell fate and the transforming cell properties of CSCs. p53 binds to the Nanog promoter, repressing its expression in conjunction with the co-repressor mSin3a. p53 is critical to pluripotent cell reprogramming and stem cell renewal [143]. In animal experiments performed by Moon et al. (2011), murine p53^−/−^ astrocytes are de-differentiated into CSC-like cells while maintaining neural stem and progenitor phenotypes and self-renewing activities, undergo neurosphere formation in vitro and form tumors in vivo [144].

Increasing evidence suggests that these diverse *mut*p53 GOF processes occur through multiple pathways. Fiore et al. (2014) demonstrated that *mut*p53 GOF causes differentiation of human osteosarcoma MG-63 cells into 3AB-OS CSCs. They investigated the expression of *wt*p53 in 3AB-OS CSCs compared with parental MG-63 cells. Parental cells contain a single copy of *wt*p53 gene, and the promoter is methylated. 3AB-OS cells contain a rearranged TP53 gene of multiple copies by the P72R polymorphism and hot spot mutation R248W. Ectopic expression of p53-R248W/P72R lends to a cancer a stem-like phenotype, possessing a high proliferative rate with clonogenic growth, undergoes sphere formation and exhibits strong features of invasiveness and migration. Stem cells and p53 have been associated with Wnt/β catenin signaling pathways, Sonic Hedgehog (Shh), BMI-1 (B-cell-specific Moloney murine leukemia virus insertion site 1), Notch and PTEN pathways that provide a delicate balance of self-renewal and differentiation through niche signaling and the interplay among these pathways [97,126,145]. When these signaling pathways become deregulated, the uncontrolled self-renewal capacity of CSCs is presented.

Accumulating evidence demonstrates that p53 loss can lead to an acquisition of stemness in cancer, highlighting the role of *wt*p53 and *mut*p53 in non-malignant somatic stem cells and CSCs. Recognizing the similarities between ontogeny and oncology, p53 deficiency and stemness may collectively contribute to tumor initiation and recurrence through survival advantages, such as: disruptions in cell death programming, drug resistance and self-renewal. In non-malignant cells, it has been shown that p53 can influence stem cell homeostasis and pluripotency. *wt*p53 has been observed to counteract somatic cell reprogramming and constrain iPSC generation in vivo [137,138,146,147,148,149,150]. A reduction in p53 increases cell reprogramming efficacy and facilitates induced pluripotent stem cell (iPSC) generation [137]. p53 may also act to regulate stem cell fate by targeting a variety of factors, such as Wig1, a transcriptional p53 target encoding a zinc-finger protein involved in post-transcriptional gene regulation, and is found to be highly expressed in stem cells [151]. In tumors, *mut*p53 acquires GOF processes to influence stemness as observed in its ability to stimulate iPSC formation while augmenting the malignant potential of reprogrammed cells [20]. p53 can suppress the epithelial-mesenchymal transition (EMT), an activator of stemness and cell motility phenotype, and activate miR-200c expression, which suppresses EMT-activators, ZEB factors and translational stem cell factors (e.g., BMI-1, Nanog, Sox2) to induce mesenchymal-epithelial-translation (MET) [152,153,154,155,156]. In fact, miR-200 family members are downregulated in breast CSCs and breast epithelial stem cells [157]. *Mut*p53 GOF induces EMT transition by modulating the miR-130b-ZEB axis in endometrial cancer [158]. These emerging links between p53 and stem cell homeostasis and pluripotency enabled Arsic et al. (2015) [24] to propose that p53 be considered the “guardian of reprogramming” in addition to its recognized role in the genome.

Like p53 mutants, p53 isoforms have also been implicated in promoting CSC potential. Overexpression of the p53 isoform p53∆133p53β promotes stemness in MCF-7 breast CSCs by regulating the expression of stem cell factors Sox2, Oct3/4, Nanog and CD24/CD44 and promoting mammosphere formation [24]. Furthermore, ∆133p53β induction through etoposide treatment enhances cancer cell stemness. Gong et al. (2015) [159] showed that p53 isoform ∆113p53/∆133p53 promotes DNA double-strand break repair to protect cells from death and senescence in response to DNA damage. p53 maintains ESC genetic stability as a DNA damage repair response by inducing differentiation. Disrupting or inactivating of p53 increases pluripotent stem cell production [146]. p53^−/−^ iPSCs exposed to DNA damage, carry short telomeres and chromosome aberrations and are genetically unstable [138]. By decreasing p53 levels in mouse fibroblasts, Kawamura et al. (2009) demonstrated that two critical transcripts, Oct-4 and Sox2, increase iPSC production [137]. p21, a known p53 target, was also found to be a main player in p53-directed reprogramming and generation of iPSCs. Ungewitter and Scrable (2010) [160] showed that ∆40p53, a transactivation-deficient isoform of p53 that is highly expressed in ESCs and during early stages of mouse embryogenesis, controls the switch from pluripotent ESCs to differentiated somatic cells. ∆40p53 acts as a master regulator of this pluripotency switch via IGF and PI3K signaling by controlling IGF-1 receptor (IGF-1R) levels and the activity of full-length p53 with Nanog and IGF-1R.

**Figure 2:**
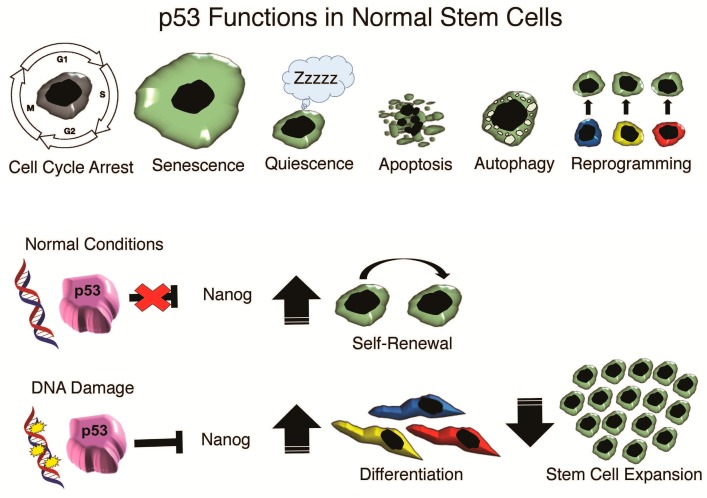
p53 functions in normal stem cells at normal conditions and DNA damage.

Accumulating evidence has linked *wt*p53, *mut*p53 and p53 isomers to stemness in both non-malignant stem cells, such as ESCs, iPSCs and tumor CSCs. It is becoming increasingly evident that p53 may possess a stemness role in non-malignant and CSCs beyond its main tumor suppressor function. p53 suppresses self-renewal and induces differentiation in embryonic development and organogenesis. In response to DNA damage, mouse ESCs (mESCs) activate p53 to bind to the promoters of self-renewal and pluripotency genes Nanog and Oct4, evoking differentiation into cell states responsive to p53-induced cell cycle arrest or apoptosis [106,107,161]. Activation of p53 through Oct4 silencing promotes human ESC (hESC) differentiation due to the repression of the p53 inhibitor Sirt1 and acetylation of p53 at lysine 120 and 164 [162]. Understanding how the mechanisms of p53 stemness function in normal and malignant cells may reveal novel targets for therapeutic intervention. Aggressive tumors with stem-like phenotypes may be prone to manipulation by directing differentiation into responsive cell types. Targeting self-renewal signaling pathways or cell surface markers could also be implemented to eradicate CSCs. Furthermore, restoration of *wt*p53 and inhibition of *mut*p53 and CSC-associated p53 isomers might lead to the reduction of drug resistance and enhance treatment efficacy (Figure 2).

## 3. p53 Gain of Function Roles in Stemness

### 3.1. Differentiation and Dedifferentiation

Studies have confirmed that p53 regulation of stem cell differentiation processes is critical in preventing malignant transformation. Studies showcasing p53^−/−^ human ESCs have confirmed the suppressive role of p53 in regulating hESC pluripotency and maintaining genomic stability [78,80,81,163]. p53 suppresses self-renewal of ESCs in response to DNA damage [164]. In experiments by Lin et al. (2005), p53 suppresses Nanog expression following DNA damage inducing mESC differentiation during oncogenic stress to inhibit cell proliferation [107]. Using hESCs, Maimets et al. (2008) [165] demonstrated that Nutlin, a small molecule inhibitor that binds to the p53 antagonist Mdm2, activated p53 to promote cell differentiation and degradation of cyclins A and E, dephosphorylation of the activating phosphorylation site of CD2 Thr-160, elimination of S phase entry into the cell cycle and activation of p21.

In cancer, loss of p53 tumor suppressor function often accompanies *mut*p53 GOFs, which enhances tumorigenesis, metastasis, drug resistance, genomic instability and multinucleation [166,167,168,169,170,171,172,173,174]. *wt*p53 loss has been associated with enhancing the efficiency of somatic cell reprogramming to a pluripotent state and stem-like phenotype in cancer. Mizuno et al. (2010) [175] reported that inactivation of p53 in breast cancers and mutations in p53 correlated with stem cell phenotype. Sarig et al. (2010) [20] reported that *mut*p53 facilitated somatic cell reprogramming and augmented the malignant potential of reprogrammed cells. Likewise, Fiore et al. (2014) [176] found that *mut*p53-R248W/P72R exhibited GOF activity in 3AB-OS MG63 osteosarcoma cells with its ectopic expression promoting aggressiveness, chemoresistance and cancer stemness. While investigating tumor progression in epithelia ovarian cancer, Ren et al. (2016) [177] reported *mut*p53 regulation of tumor differentiation and responsiveness to steroids. Furthermore, Lu et al. (2013) [178] demonstrated a *mut*p53 R175H GOF in programming mammary tumorigenesis by expanding mammary epithelial stem cells (MESCs) that generate tumors. However, the molecular mechanisms for how p53 promotes stemness and whether the stem-like cells acquire survival advantages for tumor initiation and progression are not clearly understood [174].

Oxidative stress activates p53 during tissue injury hypoxia resulting in death and differentiation of hESCs. However, hESCs are able to avoid this fate by exerting a cytoprotective effect. Das et al. (2012) reported conditional reprogramming activity or “enhanced stemness” by elevating HIF2α transcriptional activity in SSEA3^+^/ABCG2^+^ hESCs through transient suppression of p53 activity. The enhanced stemness was transient; however, it provided a highly cytoprotective state with enhanced transcriptional activity of Oct-4 and Nanog in undifferentiated cells. The hESCs also demonstrated high Bcl-2, fibroblast growth factor 2 (FGF2), altering the oscillation system state of p53/Mdm2 [179]. Itahana et al. (2016) [180] showed in hESCs that histone modifications and p53 binding enable p21 promoter activation. hESCs’ high proliferation rate is due to low expression of p21. p53 binds to the p21 promoter of hESCs, but does not promote p21 transcription in hESCs, as it does in differentiated human mesenchymal stem cells (hMSCs). Enrichment of repressive and activating histone H3K27me3 at the p21 locus in hESCs overrides activation by p53. Therefore, H3K27me3 methylation rescues p21 expression while p21 ectopic expression induces hESC differentiation. Epigenetic signals to hESCs prevent undesired p53 target genes from initiating differentiation and maintain genomic stability and homeostasis.

### 3.2. Epithelial-Mesenchymal Transition and Stemness

EMT is a reversible process in which polarized epithelial cells lose their cell-cell adhesions and acquire a spindle-like morphology. The accompanying mesenchymal properties enable cells to migrate and invade distant tissues. This process is essential for gastrulation, neural crest delamination of vertebrates, tissue formation, embryonic cell development, wound healing, fibrosis, carcinogenesis and metastasis [181,182,183]. To initiate metastasis, cancer cells activate EMT through environmental signals inducing the expression of SNAIL, TWIST, ZEB1/2, SLUG, SMUC, bHLH factor E47 and BMI-1 transcription factors that interact with epigenetic regulators. Many of these transcription factors target the CDH1 gene, which encodes a fundamental EMT protein, E-cadherin. This trans-differentiation program is driven by a complex network of protein and gene regulation including N-cadherin and *vimentin* induction, cytoskeletal changes, cell adhesion and ECM degradation [181,184,185]. Studies associated the induction of EMT to acquire CSC molecular and functional traits [186,187]. In immortalized or transformed human mammary epithelial cells, the overexpression of TWIST, ZEB1 or SNAIL converts the cells from a differentiated profile (CD44low/CD24high) to a breast CSC signature (CD44^+^/CD24^−/low^), enabling tumorsphere formation [141,186,188]. Inhibition of p53 combined with mitogenic oncoproteins, serving as EMT-inducing factors, drives tumorigenesis, cancer stemness and cell plasticity. Spike et al. (2011) associated p53 activity with stemness modulation in embryonic and undifferentiated cells [174]. During embryogenesis, p53 maintains embryonic cell proliferation and preserves stemness [189,190]. Cicalese et al. (2009) [191] demonstrated that p53 was able to regulate the polarity of self-renewing divisions in mammary stem cells. Loss of p53 increased CSC symmetrical divisions, mammosphere formation, tumor initiation and tumor growth. Suppression of p53 inhibits stem cell self-renewal and the reprogramming quality of differentiated cells into iPSCs [107,192,193,194]. In cancer, p53 inactivation is associated with EMT and cancer stemness [174,175,195]. TWIST1 is upregulated in a *mut*p53 background promoting the induction of SLUG expression [196,197].

Chang et al. (2011) [152] showed that p53 can regulate EMT and stem cell properties by modulating miRNAs. This study found that p53 transactivated miR-200c by directly binding to the miR-200c promoter. Loss of p53 in mammary epithelial cells decreased miR-200c expression to activate EMT and increased mammary stem cell numbers. *wt*p53 directly activates transcription of miR-200 and miR-34 gene loci, creating reciprocal feedback loops to reduce EMT inducers ZEB1 and SNAIL [152,198,199,200]. miR-200 and miR-34 directly inhibit the translation of stem cell factors BMI1, CD44 and CD133 [157,201,202]. p53 can also activate miR-145 expression to repress stem cell factors Oct4, KLF4, LIN28A and Sox2 [203]. Korpal et al. (2008) [156] reported that miR-200 and miR-194/192 repress ZEB1/2 expression to negatively regulate EMT and metastasis. *wt*p53 directly activates the miR-200c and miR-192 transcription leading to ZEB1/2 downregulation and EMT repression [200]. *Mut*p53 promotes EMT and tumor aggressive potential by inhibiting *wt*p53-miR-200c pathways via dominant-negative effects on *wt*p53 [152]. Dong et al. (2013) [158] demonstrated that a *mut*p53 GOF facilitates EMT and cancer cell invasion through repression of the ZEB1 inhibitor, miR-130b. Shi et al. (2014) [204] reported that p53-induced miR-15a/16-1 and AP4 form a double-negative feedback loop to regulate EMT and metastasis in colorectal cancer. These findings support the role of p53 to function in cellular plasticity, EMT and stemness via regulation of miR-200, miR-34, miR-145 and miR-15a/16.

### 3.3. p53 and Stem Cell Senescence and Quiescence

In response to genotoxic stress, the tumor suppressor p53 induces transient growth arrest or apoptosis and mediates a cellular process of limited proliferation and irreversible growth referred to as senescence and the cell cycle G1/S checkpoint. p53 isoforms have also been implicated in replicative senescence and, similar to *wt*p53, participate in cell cycle progression, angiogenesis, programmed cell death, viral duplication and cell differentiation [205,206,207,208,209,210]. Cells with significant DNA damage undergo p53-dependent apoptosis [211]. Sugrue et al. (1997) [212] demonstrated that *wt*p53 triggers senescence in human tumor cells lacking functional p53. Dividing and proliferating cells frequently experience more DNA double-strand break damage than do non-proliferating cells [213,214]. mESCs do not experience p53-dependent apoptosis at the G1/S checkpoint after DNA double-strand break damage and, therefore, do not experience cell cycle arrest, apoptosis and senescence. p53 can suppress the ESC self-renewal and pluripotency after DNA damage [215]. p53^−/−^ hESCs have been used to show that the role of p53 in suppressing pluripotency is conserved in mouse and human ESCs. In MEFs, Ferbeyre et al. (2002) reported that oncogenic *ras* and p53 cooperate to induce senescence through activation of the MAP kinase pathway [216]. Gannon et al. (2011) [217] showed that mice lacking Mdm2 in the epidermis activate p53 signaling in the epidermal stem cell to promote senescence and premature aging phenotypes in mouse skin as characterized by thinning of the epidermis, reduced wound healing and progressive loss of fur.

**Figure 3:**
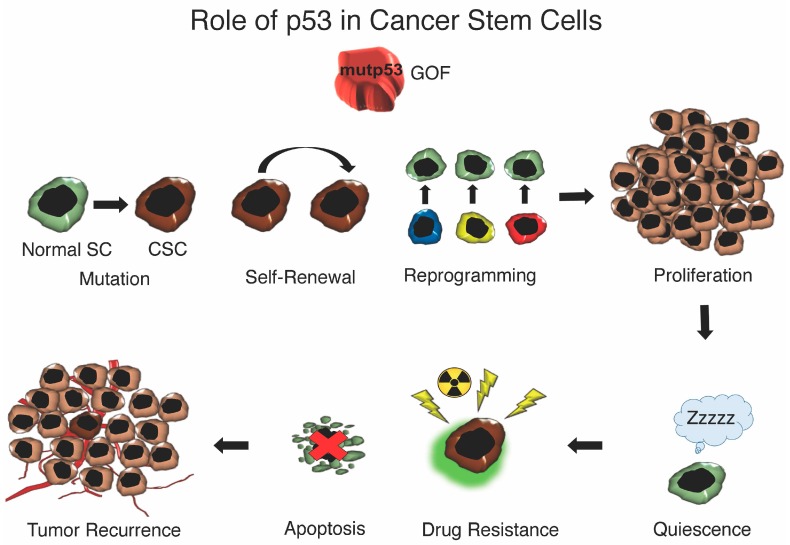
Role of p53 in cancer stem cells.

Contemporary chemotherapies can obliterate the majority of dividing and proliferative cancer cells in the tumor; however, the inability to completely eradicate CSCs ensures tumor recurrence (Figure 3). CSCs have been shown to acquire resistance mechanisms, such as DNA repair, drug efflux, ATP-binding cassette (ABC) transporters, detoxifying agents, anti-apoptotic agents, morphological changes and quiescence [218,219,220]. Quiescence or slow cell cycling is a feature shared among non-malignant stem cells and CSCs involved in self-renewal and preventing stem cell exhaustion. Quiescence is a reversible process that can be restored by stimulation with the addition of growth factors to resume proliferation. HSCs likely use quiescence to maintain the HSC self-renewal compartment for the lifetime of the organism to sustain and give rise to all hematopoietic lineage cells [221,222,223,224]. Quiescence also protects dormant stem cells from various stresses, such as myelosuppression induced by 5-fluorouracil (5FU)-treatment. Similarly, CSC are able to avoid the effects of chemotherapy by acquired resistance [221].

In hair follicle stem cells (HFSCs), the state of quiescence is a form of tumor suppression. Cancer cells originating from HFSCs give rise to cutaneous squamous cell carcinoma. Tumorigenesis is averted when the cell cycle is stalled at G0/G1, suggesting that the processes maintaining HFSCs’ dormancy are dominant over oncogene gain (i.e., Ras) or p53 tumor suppressor loss. Many intrinsic mechanisms known to regulate quiescence include transcription factors FoxO, HIF-1α and NFATc1, and signaling through ATM and mTOR. Multiple extrinsic regulatory mechanisms in the microenvironment have been identified, including bone morphogenic protein (BMP), osteopontin, thrombopoietin (TPO), angiopoietin-1 (Ang-1), tumor growth factor-β (TGF-β), N-cadherin and integrins, as well as Wnt/β-catenin signaling [225]. PTEN is an alternative factor that contributes to the maintenance of quiescence in the presence of tumorigenic stimuli preventing tumorigenesis [226]. PTEN also plays an important role in sustaining p53 levels in tumor cells, which may concomitantly regulate stem cell quiescence. In U87MG glioblastoma, PTEN protected p53 through inhibition of phosphophatidylinositol 3-kinase (PI3K)/Akt signaling (activation known to promote Mdm2 translocation into the nucleus), resulting in Mdm2 restriction to the cytoplasm, where it is degraded. As a result, p53 levels and transactivation increase, sensitizing U87MG glioblastoma cells to DNA damage and p53-mediated cell death induced by the chemotherapeutic agent etoposide [41]. Furthermore, the PTEN gene is a transcriptional target of p53, and p53 selectively targets PTEN over the Mdm2 gene in cells with sustained genotoxic stress [41]. Though not explored in these studies, it is possible that a chemotherapy response could regulate the PTEN-p53 axis and CSC quiescence.

p53 is essential for restraining cell cycle entry. Many studies have associated p53 with regulation of stem cell quiescence. Loss of p53 in neural stem cells (NSCs) and HSCs triggers stem cell expansion, as these cells exit quiescence and progress through the cell cycle [227,228]. Furthermore, Cheng et al. (2013) [229] demonstrated that conditional deletion of Cdkn1a, a p53 target gene encoding cyclin-dependent kinase inhibitor p21, leads to NSC and HSC stem cell proliferation and exhaustion. Liu et al. (2009) [230] showed that p53 regulates HSC quiescence and enhanced quiescence in HSCs lacking the MEF/Elf4 transcription factor, which is known to regulate both HSC self-renewal and quiescence. HSCs transcriptional profiling of *wt*p53 and p53 null mice revealed Gfi-1 and Necdin to be p53 target genes. Gfi-1 maintains HSC functional integrity and inhibits HSC proliferation, while Necdin is a negative cell cycle regulator increasing quiescence.

Itahana et al. (2002) [227] reported that p53 contributes to a reversible, growth factor-dependent arrest of quiescence (G0) in normal WI-38 human fibroblasts. To abrogate p53 function, fibroblasts were microinjected with Mdm2 or the pRb binding mutant of SVT antigen plasmid DNA, which stimulated quiescent fibroblasts to initiate DNA synthesis. p53 activity was higher in quiescent and senescent fibroblasts compared to actively-proliferating cells when analyzed by electrophoretic mobility shift and p53 transactivation. Growth factor withdrawal from proliferating cells led to G0 arrest, elevated mRNA levels and accumulation of p53, followed by an increase in p21 mRNA and protein expression. p21 is a p53 target gene and effector of cell cycle arrest. Furthermore, stable expression of p53 inhibitors HPV16 E6 oncogene and dominant negative p53 peptide (GSE-22) delayed entry into G0 after growth factor withdrawal [227]. Brien and Bracken (2016) [231] described the polycomb like 1 (PCL1)-p53 regulatory axis as important for the maintenance of cellular quiescence. During development and differentiation, the chromatin-associated transcriptional repressor polycomb proteins maintain cellular identity. The PCL1 gene promoter is a direct transcriptional target of p53 with the N-terminal PHD domain of PCL1 protein binding to the C-terminal regulatory domain (CTD). This interaction stabilizes p53 and activates CDKN1A to promote quiescence [229,232].

## 4. Therapeutic Strategies to Restore *wt*p53 Function in CSCs

### 4.1. Small and Large Molecules

Molecule development to explore novel targets and signaling pathways to restore *wt*p53 function is necessary for probing the mechanisms of p53 inactivation in CSC genesis and tumor development. Loss of *wt*p53 function is prevalent in more than half of all solid and hematologic malignancies and persists as mutations or deletions altering p53 activity levels. *Wt*p53 found in cancers is nonfunctional in that p53 is either degraded by Mdm2, or secluded from the nucleus inhibiting its function in transcription [34,233,234]. Changes in epigenetic regulation of p14/ARF, methylation of the p53 promoter and persistent expression of Mdm2 and Mdmx contribute to *wt*p53 loss of function [136].

**Figure 4:**
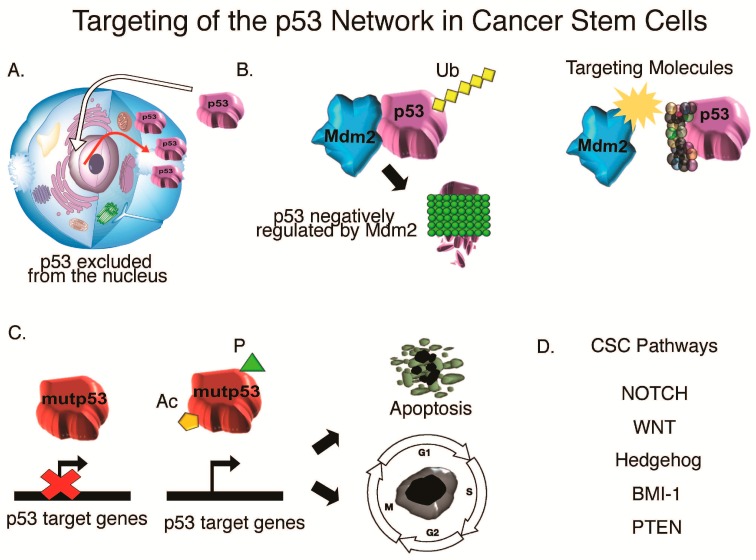
Targeting of the p53 Network in cancer stem cells. (**A**) Restoring p53 transcriptional activity be preventing/restoring p53 exclusion from the nucleus; (**B**) targeting molecules to disrupt the p53-Mdm2 interaction to prevent p53 degradation; (**C**) restoring mutant p53 function by modifications; (**D**) targeting CSC pathways.

Combinatorial therapies have been employed to target *mut*p53 in CSCs and improve therapeutic strategies, including chemotoxic agents (Figure 4). Many of these small molecules have been shown to be effective in preliminary studies against solid tumors and hematological cancers. These molecules can be classified as *wt*p53 activators (RITA, Nutlin-3, MI-219, BDA, HL198C, Tenovin-1, JJ78:12), *mut*p53 reactivators (CP-31398, PRIMA-1, MIRA-1, ellipticine, P53R3, WR-1065) or *wt*p53 inhibitors (PFT-α, PFT-µ) [235,236,237,238,239,240,241,242,243,244,245,246,247,248,249].

Huang et al. (2009) used Ellipticine with 5-FU as a strategy to deplete deficient p53 DLD1 colon CSC. The use of small molecules, such as CP-31398, MIRA1 or PRIMA-1, to induce transactivation activities in *mut*p53 has also been employed [243,250,251,252]. PRIMA-1 and MIRA-1 have unique chemical structures, but both share the ability to restore the p53 conformation from mutant to wildtype [242,243]. PRIMA-1 converts to a methylated form, PRIMA-1^MET^, and modifies thiol groups in the central domain of *mut*p53 to restore tumor suppressor p53 [253]. Zhang et al. (2016) [250] used PRIMA-1 to target *mut*p53 in CSC from breast, endometrial and pancreatic cancer cell lines, allowing for the reduction in tumor cell growth and inhibition of tumorsphere formation. In solid tumors, small molecule MIRA-1 can induce apoptosis through p53-dependent processes [243]. The p53 activity of chemoresistant colorectal CSCs can be restored by the small molecule Prodigiosin through c-Jun-mediated ∆Np73 downregulation and p73 activation [254].

Synthetic peptides derived from known p53 binding proteins can restore the conformation of *mut*p53 to *wt*p53, including the transcriptional activity of hotspot p53 mutants p53R273H and p53R175H, such as peptide CDB3 with l-amino acid sequence REDEDEIEW [255]. Peptide 46, a p53 C-terminal peptide that increases tumor efficacy through core domain stabilization leading to p53 reactivation and apoptosis [256,257,258]. Stapled peptides or stitched peptides have been retrofitted with a synthetic brace to promote peptides stabilizing an alpha-helix structure that disrupts the p53-Mdm2 interaction and reactivating the p53 tumor suppressor pathway [259]. Positively charged cell-permeable stapled peptides have also been shown to inhibit the p53-Mdm2/Mdmx complex [260]. These peptides can be combined with other targeting approaches, such as polymeric micelles for enhanced tumor penetration and access to the blood brain barrier (BBB) [261]. Bispecific antibodies have also shown promise in targeting the p53-Mdm2 interaction by binding to p53. Weisbart et al. (2004) [262] demonstrated that a bispecific single-chain antibody can penetrate colon cancer cells with *mut*p53 and restore *wt*p53 function.

### 4.2. RING Finger E3 Ligases and CSCs Maintenance

Recent studies have discovered RING finger E3 ligases to be important contributors to cell stemness via regulation of self-renewal, differentiation and drug resistance displayed by CSCs. Inhibition of RING finger proteins may provide promising strategies for cancer treatment. RING finger proteins are not catalysts with active sites; instead, they facilitate ubiquitin transfer from an E2 directly to the substrate. Disrupting the RING structure or RING-E2 interface would represent viable routes for inhibitor development. Nutlin-3 is a small molecule antagonist, which binds to the amino terminus of Mdm2 and prevents binding to p53, leading to increased stability of p53 [236]. As a result of stabilizing p53, cancer cells are able to undergo classic p53-mediated cell cycle arrest and apoptosis. Kang et al. (2014) [263] reviewed the regulation of CSCs by RING finger ubiquitin ligases and reported that SKP1-CUL1-F-box protein (SCF) E3s, CBL, BRACA1, MDM2 and von Hippel–Lindau tumor suppressor (VHL) are crucial in the regulation of cell-cycle progression, cell differentiation and CSC maintenance.

As the guardian of the genome, p53 maintains cellular integrity by inhibiting the cell cycle and preventing the proliferation of damaged cells [264]. These processes are controlled via negative regulation of the tumor suppressor p53. There are many ubiquitin ligases that can regulate p53, Mdm2, Pirh2, Cop1, Trim24, ArfBP1, Carp1/2 and TOPORs. While some of these ligases are associated with CSCs, their role with respect to tissue-specific regulation of p53 or even stimuli-specific regulation of p53 remains to be determined.

ESCs and early embryos endogenously express high levels of full-length p53 and remain viable upon removal of p53 [13,265,266,267]. However, mice lacking Mdm2 succumb to early embryonic lethality and expire before implantation. Active p53 leads to embryonic lethality in Mdm2-deficient mice, but interestingly, the phenotype can be rescued by concomitant deletion of p53, demonstrating that negative regulation of p53 is essential during early embryogenesis and for ESC survival [268]. In HSCs and progenitors, loss of Mdm2 activity stabilizes p53 and impedes hematopoiesis via induction of cell cycle arrest, senescence and cell death [176]. Mdm2 is overexpressed in many high grade cancers with over a third present in tumors with *wt*p53 [269]. The MDM2 gene is transcriptionally regulated by p53, and alternative splicing can give rise to several Mdm2 transcript variants expressed in cancer. Mdm2 can also bind to tumor suppressor ARF with the interaction resulting in sequestration of Mdm2 in the nucleolus, where it cannot bind and degrade p53, therefore activating p53.

Researchers have embarked on associations of Mdm2 with stemness in a variety of cellular processes. Daniele et al. [270] (2015) demonstrated that a combined inhibition of Mdm2 and Mdm2 stimulating the AKT/mTOR pathway using inhibitors ISA27 and FC85, respectively, enhanced glioblastoma multiforme (GBM) cell apoptosis and differentiation of CSCs. In MEFs, Wienken et al. (2016) [271] demonstrated that efficient iPSC generation is dependent on Mdm2 in absence of p53. While in hMSCs, Mdm2 depletion promoted cell differentiation and decreased clonogenic survival in cancer. Inactivation of the Polycomb Repressor Complex2 (PRC2) and EZH2 can induce many genes regulated by Mdm2. The complex formation of Mdm2 chromatin and EZH2 increased trimethylation and ubiquitination of histone 2 (lysine 27) and histone 2A (lysine 119; H2AK119), respectively. Furthermore, the absence of Mdm2 and H2AK119 E3 ligase Ring1B/RNF2 inhibited cell proliferation. Independent of p53, Mdm2 is associated with inhibition of lineage specific genes via the polycomb [271].

In patients with newly-diagnosed glioblastoma, temozolomide (TMZ) is the standard of treatment; however, a challenge exists in overcoming TMZ drug resistance mainly due to O^6^-methylguanine DNA methyltransferase (MGMT). Removal of the methyl group from TMZ induces chemoresistance in glioma stem cells and is prevalent in a significant fraction of glioblastoma cases. Sato et al. (2011) [272] show that the mitogen-activated protein/extracellular signal-regulated kinase (MEK)-extracellular signal-regulated kinase (ERK)-Mdm2-p53 pathway regulates MGMT expression using patient-derived stem-like glioblastoma cells. Mdm2 was found to be upregulated via the MEK-ERK signaling pathway, thus inhibiting p53 function and maintaining the expression of MGMT. This study was pivotal in demonstrating that MEK inhibition could render resistant CSCs sensitive to TMZ. Dual treatment with a MEK inhibitor and TMZ can effectively control glioma CSCs from initiating tumors.

Additionally, Abbas et al. (2010) [176] reported HSC/progenitor survival to be dependent on Mdm2 reduction of p53 activity induced by reactive oxygen species (ROS). In response to ROS, p53 trans-activates genes that regulate proliferation, differentiation, senescence and apoptosis [273]. It has been reported that p53 can elevate ROS by activating ROS-inducing genes Pig1, 8 and 12 [274]. In contrast, p53 has also been reported to protect stem cells from ROS damage via upregulation of GOX, p53-induced genes (PIGs) and downregulation of nitric oxide synthase 2 (NOS2) and cyclooxygenase 2 (COX2) [176]. While implementing a p53 mutation capable of inducing cell cycle arrest without apoptosis, Mdm2^−/−^ p53 515C (encoding p53R172P) mice were generated resulting in normal HSC counts in fetal livers and depleted counts in postnatal bone marrow. These mice are also observed with increased ROS levels and p53R171P expression, activation of p16 lnk4a and cell death. The experimental study signifies the importance of the p53-Mdm2 pathway in hematopoiesis. In the model, basal ROS levels accumulate in hematopoietic tissues during development. The absence of Mdm2 in combination with basal oxidative stress permits p53 activity to induce ROS production via transactivation of PIGs, creating a positive feedback loop. HSCs and progenitors become depleted via the induction of cell cycle arrest, senescence and cell death. Sensitization of bone marrow HSCs to the ROS-p53 pathway can be exploited in leukemia, because leukemia stem cells can propagate from HSCs. During normal hematopoiesis, stress signals activate p53, and Mdm2 is required for p53 level regulation to enable stem cell survival [275].

## 5. Discussion

Stem cells serve an indispensable role in normal development and organ regeneration. Stem cells and progenitors can differentiate into various tissues in the body, repair damaged tissue and serve as a resident population of quiescent cells throughout the life of an organism. However, these cells may undergo neoplastic transformation, leading to dysregulation in normal stem cell processes. Uncontrolled self-renewal and differentiation result in deleterious consequences on development, aging and tumorigenesis. p53 is involved in several distinct processes that influence normal stem cell functions, maintaining stem cell number and quality.

Functioning as a guardian between non-malignant stem cells and CSCs, p53 prevents stem cell dedifferentiation and propagation of cells with genomic abnormalities. Many advances have been made in unraveling the mechanisms of cancer progression. The tumor suppressor p53 has been an investigative focal point for nearly four decades. Despite the advances in current cancer therapeutics, advanced tumors continue to evade our most versatile treatments due to the inability to effectively target and eliminate CSCs. It is now evident that a link has been revealed between the concept of “stemness” and its regulation by the guardian of the genome, p53, in non-malignant stem cells and CSCs.

In order to adequately treat cancer and ensure the success of President Barack Obama and Vice President Joe Biden’s 2016 National Cancer Moonshot Initiative, it is imperative that future studies focus on understanding the mechanisms that maintain stable genomes in stem cells and how these signaling pathways are deregulated in the genesis of CSCs and CSC-mediated tumor recurrence. Studies have revealed similar signaling pathways among non-malignant stem cells and CSCs. The Wnt, Hedgehog, BMI-1, Notch and PTEN evolutionarily-conserved pathways have demonstrated regulatory roles in the self-renewal and the cell fate decision of embryonic and hematopoietic stem and progenitor cell, as well as the maintenance of CSCs. *wt*p53, p53 isomers, *mut*p53 and p53 binding and regulatory factors, such as Mdm2, have been implicated in the stemness regulation processes. Targeting these CSC pathways and their intricate and interactive tumor milieu may prevent tumor recurrence following surgery, chemotherapy and radiation.

p53 isomers encompass at least 12 different p53 proteins, and *mut*p53 constitutes a multitude of proteins. Most investigations of *mut*p53 have been explored at mutations in the amino acids 175, 245, 248, 249, 273 and 282 in the DNA binding domain. Likewise, other domains have been found to be mutated, but their contribution to tumorigenesis and stem cell regulation have not been thoroughly investigated [276]. Different tumor types at varying stages of malignancy display a diverse spectrum of *mut*p53 protein function in a variety of oncogenic and stem-conferring processes. The frequency of missense mutations differs in subclasses of tumors of the same organ. This is evident in breast cancers where luminal types mostly exhibit p53 point mutations, whereas basal tumors mostly detect alternations resulting in p53 truncation [277]. Understanding the distinct functions of each form of p53 protein is central to discovering the stemness mechanism that enables CSCs to propagate and interact with specific tumor niches and promote metastasis. Developing novel drug strategies for these p53 targets influencing tumorigenesis and cancer stemness will be challenging and necessitate a critical understanding of the p53, p53 isomers and *mut*p53 upstream and downstream stem pathways and interacting partners, such as PTEN and MDM2.

When investigating the processes contributing to stemness, it is vital to understand which factors come into play. The frequencies of CSCs were once believed to be as rare as 0.0001% to 0.1% of all cancer cells [79,85]. Recent advances in our understanding of factors contributing to CSCs have allowed for frequencies to be defined as cell type dependent, noting prominence in cancer types, such as melanoma and colorectal carcinoma [76,78,278]. Specific xenotransplantation assays can influence the outcome of the percentage of cells that induce tumorigenesis by several orders of magnitude. While it has been reported that Mdm2 overexpression is associated with CSCs, there are several underlying questions regarding how CSCs induce the *Mdm2* gene. How are Mdm2 protein functions dependent on activated signaling pathways and the many Mdm2 isoforms generated from alternative start sites or mRNA splicing? How does Mdm2 switch from ubiquitinating to a neddylating E3 in CSCs? Thus, it is imperative to completely understand how Mdm2 is regulated and how it regulates stemness as targeting Mdm2 to prevent CSCs may inadvertently promote CSC stemness and tumor heterogeneity. Thus, the complexity of p53 gene splicing and mutations and its regulatory network is currently evolving in the context of CSC, and further studies are necessary to gain critical information on how CSC may or may not respond to p53-targeted therapy.

## Abbreviations

5-FU	5-Fluorouracil
ABC	ATP-binding cassette
ADR	Adriamycin
AKT/mTOR	Protein kinase B or serine/threonine-specific protein kinase/mechanistic target of rapamycin
Ang-1	Angiopoietin 1
ATP	Adenosine triphosphate
Bcl-2	B-cell lymphoma 2
bHLH factor E47	Basic helix-loop-helix factor E47
BMP	Bone morphogenetic protein
BBB	Blood brain barrier
BRACA1	Breast cancer gene 1
CAF	Cancer-associated fibroblast
CBL	Casitas B cell lymphoma
CDKN1A	Cyclin-dependent kinase inhibitor 1A
COX	Cyclooxygenase2
CSC	Cancer stem cell
CTD	C-terminal regulatory domain
DNA	Deoxyribonucleic acid
H2AK119 E3 ligase	Histone 2A at lysine 119 E3 ligase
hESC	Human embryonic stem cell
ECM	Extracellular matrix
ERK	Extracellular signal-regulated kinase
EMT	Epithelial-mesenchymal-transition
FoxO	Forkhead box O
FAK	Focal adhesion kinase
Gfi-1	Growth factor independent 1 transcription repressor
GOF	Gain-of-function
GOX	Glucose oxidase
HIF1	Hypoxia-inducible factor 1
HIF2	Hypoxia-inducible factor 2
HFSC	Hair follicle stem cell
HMGA2	High mobility group at-hook 2
HSC	Hematopoietic stem cell
ICM	Inner cell mass
iPSC	Induced pluripotent stem cells
KLF4	Krüppel-like factor 4
LIN28A	Lin-28 homolog A
MAPK	Mitogen-activated protein kinase
mCSC	Metastatic cancer stem cells
Mdm2	Mouse double minute 2 homolog
MEFs	Murine embryonic fibroblasts
MEF/Elf4	Myeloid Elf-1-like factor/ETS-related transcription factor 4
MEK	Mitogen-activated protein/extracellular signal-regulated kinase kinase
mESC	Mouse embryonic stem cell
MET	Mesenchymal-epithelial-transition
MGMT	*O*^6^-methylguanine DNA methyltransferase
MSC	Mammary stem cell
Mut p53	Mutant p53
Nedd4	Neural precursor cell expressed developmentally down-regulated protein 4
NOD/SCID	Non-obese diabetic/severe combined immunodeficiency
NOS2	Nitric oxide synthase 2
NOTCH	Notch homolog 1, translocation-associated
NSC	Neural stem cells
PCL1	Polycomb like 1
PHD	Plant homeodomain
PI3K/Akt	Phosphatidylinositol-3-kinase
PIGs	p53-induce genes
PRC2	Polycomb repressor complex 2
PTEN	Phosphatase and tensin homolog
pCSC	Primary cancer stem cell
rCSC	Recurrence cancer stem cell
RING	Really interesting new gene
Ring1B/RNF2	Really interesting new gene 1B/ring finger protein 2
RNAi	Ribonucleic acid interference
ROS	Reaction oxygen species
SCF	SKP1-CUL1-F- box protein
SLUG	SNAI2 or Snail family zinc finger 2
SNAIL	SNAI1 or Snail family zinc finger 1
SMUC	SNAI3 or Snail family zinc finger 3
SMWC	Small molecular weight compounds
SPARC	Secreted protein acidic and rich cysteine
TAD	N-terminal transactivation domain
TGF-B	Transforming growth factor beta
TIC	Tumor-initiating cell
TMZ	Temozolomide
TPO	Thrombopoietin
TRIM24	Tripartite motif-containing 24
TWIST1	Twist family BHLH transcription factor 1
TWIST2	Twist family BHLH transcription factor 2
USP7	Ubiquitin-specific-processing protease 7
VEGF	Vascular endothelial growth factor
VHL	von Hippel–Lindau
Wt p53	Wild-type p53
ZEB1	Zinc finger E-box binding homeobox 1
ZEB2	Zinc finger E-box binding homeobox 2
